# Risk factors associated with dengue and chikungunya seroprevalence and seroconversion among urban populations in western and coastal Kenya

**DOI:** 10.1371/journal.pntd.0013740

**Published:** 2025-11-24

**Authors:** Bethel Alebel Bayrau, Caroline Ichura, Amna Tariq, Christabel Achieng Winter, Jael Sagina Amugongo, Victoria Atieno Okuta, Laura Wanjala Mwambingu, Kevin Onyango Ogamba, Karren Nyumbile Shaita, Charles Ochieng Ronga, Philip Chebii, Said Lipi Malumbo, Omar Katana Godana, Zainabu Jawa Jembe, Charles M. Ng’ang’a, Mwangosho Mazera Mshahame, Esra Buyukcangaz, Eleonora Migliore, Donal Bisanzio, Bryson Ndenga, Francis Mutuku, Angelle Desiree LaBeaud

**Affiliations:** 1 Department of Pediatrics, Stanford School of Medicine, Stanford, California, United States of America; 2 Centre for Global Health Research, Kenya Medical Research Institute, Kisumu, Kenya; 3 Vector-Borne Diseases Unit, Msambweni County Referral Hospital, Msambweni, Kwale, Kenya; 4 RTI International, Washington, District of Columbia, United States of America; 5 Department of Environment and Health Sciences, Technical University of Mombasa, Mombasa, Kenya; Faculty of Science, Ain Shams University (ASU), EGYPT

## Abstract

**Background:**

Dengue virus (DENV) and chikungunya virus (CHIKV) are arboviruses that are endemic to Kenya. Most DENV infections are asymptomatic resulting in underreporting of cases and symptomatic cases are often misdiagnosed as malaria. Past studies focusing on arboviruses in Kenya are mostly limited to outbreak periods, leaving a gap in knowledge about inter-epidemic arboviral prevalence and associated risk factors. In this study, we aim to determine the risk factors for seroprevalence of and seroconversion to DENV and CHIKV among urban populations in two sites in Kenya.

**Methodology/Principal Findings:**

In this prospective cohort study, 4,529 participants were recruited by household from two urban sites in Kenya: Kisumu in the west and Ukunda in the coast. Participants were followed from December 2019 until February 2022 at 6-month intervals. Questionnaire data and blood samples were collected for demographic and serologic data, respectively. If a participant had a febrile illness during the study, they were registered for a sick visit, treated and blood samples were taken to test for acute DENV or CHIKV infection by RT-PCR. Our results showed a 22.8% (1,033/4529) seropositivity rate for DENV and a 21.4% (969/4,529) seropositivity rate for CHIKV; 9% (409/4529) were found to be seropositive for both. DENV and CHIKV seropositivity was more common on the coast (43.9% vs. 6% with p < 0.01for DENV, 22.6% vs. 20.5% with p = 0.09 for CHIKV) than in the west and among adults than children (30.8% vs 11.5% with p < 0.01 for DENV, 32.4% vs 5.9% with p < 0.01 for CHIKV). Of the total participants, 4% (183/4529) and 3% (136/4529) seroconverted for DENV and CHIKV, respectively, during the 2-year study period. In our multivariate analysis, controlling for variables in a stepwise selection, being from the coastal site and of older age were the main risk factors for DENV seropositivity while being from the coastal site, having greater levels of education, and crowding in the household were significant risk factors for CHIKV seropositivity. In those participants who were newly exposed to these viruses during the study period, being from the coastal site, high socioeconomic status (SES), and not having window screens in the household were the significant risk factors for both DENV and CHIKV seroconversion.

**Conclusions/Significance:**

Our results show significant DENV and CHIKV seropositivity among adults and children in urban western and coastal Kenya and evidence of active circulation of both DENV and CHIKV between 2019 and 2022. There were higher rates of seropositivity and active circulation on the coast where past outbreaks have occurred. Although lower education and socioeconomic status (SES) were reported as risk factors for arboviral infections in the past, we found more risk of seropositivity among individuals with higher SES and education, demonstrating the community-wide risk of seropositivity in urban settings. Our findings highlight the need for active surveillance of arboviruses and interventions in Kenya, especially on the coast.

## Author summary

Dengue (DENV) and chikungunya (CHIKV) viruses remain understudied in the African continent despite being major public health threats. We conducted a longitudinal prospective cohort study to determine the magnitude of DENV and CHIKV seroprevalence and seroconversion among urban populations in Kenya and assessessed individual risk factors. Participants were followed for two years at 6-month intervals and at any time during febrile illness. We found significant seropositivity for both viruses among our population with a seroprevalence of 23% for DENV and 21% for CHIKV. During the time of the study, 4% and 3% of our participants were newly exposed to DENV and CHIKV, respectively, showing active transmission of these viruses in urban settings in western and coastal Kenya. Residing on the coast and older age were significant risk factors for DENV seropositivity while older age, higher education, and having crowding in the household were risk factors for CHIKV seropositivity. Living on the coast, being of higher socioeconomic status, and having no window screens in the house were associated with seroconversion for both viruses. There should be more efforts to actively surveil arboviruses in Kenya, raise awareness about risk factors for seropositivity, and promote vector control measures to reduce disease burden and transmission.

## Introduction

Dengue virus (DENV) and chikungunya virus (CHIKV) are arboviruses transmitted by *Aedes spp.* mosquitoes that cause disease in humans [1]. DENV belongs to the genus *Flavivirus* of the *Flaviviridae* family and mostly causes asymptomatic infections [1,2]. In those exhibiting symptoms, DENV can cause fever, headache, body aches, nausea, rash, or even hemorrhagic manifestation and shock in severe cases that can be fatal [2]. CHIKV belongs to the genus *Alphavirus* of the *Togaviridae* family and can cause symptoms including characteristic severe joint pain that can be debilitating, fever, joint swelling, muscle pain, nausea, fatigue, and rash [3]. Since their symptoms overlap with each other and many other vector-borne diseases such as malaria, both DENV and CHIKV are easy to misdiagnose, contributing to the underestimation of their disease burden [3,4]. The vectors that transmit these viruses, *Aedes spp.* mosquitoes are tropical and subtropical mosquitoes native to Sub-Saharan regions of Africa, including Kenya [5]. *Aedes spp.* mosquitoes have a diurnal feeding pattern. They feed on humans during the daytime, rest in indoor habitats, and breed in containers containing still water [5]. With an increase in human movement, urbanization, environmental pollution, insecticide resistance, and global warming, *Aedes spp*. mosquitoes have expanded their spatial range, contributing to increased transmission of DENV and CHIKV in Africa [4]. Moreover, although arboviral infections are known to be prevalent in dense urban centers, recent studies have shown more seroprevalence and seroconversion of DENV and CHIKV in less densely populated areas and rural communities [6–9].

The transmission dynamics, associated risk factors, and epidemiological characteristics of DENV and CHIKV are well-studied and documented in the Americas and Asia, but they remain understudied in the African continent, where both these viruses are endemic [4,10,11]. However, few studies show a comparable burden of DENV and CHIKV in Africa compared to the Americas and Asia, with an estimated 70% of the African population at risk for arboviral disease [4,5]. Attention to such medically important arboviruses in sub-Saharan Africa has been predominantly limited to times of local outbreaks [11]. Although the majority of these sporadic outbreaks are documented, multiple studies have shown continued transmission of DENV and CHIKV in Kenya in between outbreaks that often go unrecognized and under-reported [12–16]. For example, a study conducted in Mombasa, on the coast of Kenya, enrolled febrile patients between 2016 and 2017 and reported a seropositivity rate of 74% for DENV during the outbreak period from April to March 2017 and 55% during the non-outbreak period [12]. Other flaviviruses have been detected in Kenya such as West Nile Virus, Yellow Fever, and Zika although with lower levels of incidence than DENV [17–19].

The limited study of DENV and CHIKV transmission and associated risk factors in Kenya has been attributed to many reasons. A large number of acute febrile illnesses are misdiagnosed as malaria without parasitological confirmation [20–23]. Another reason has been the limited capacity of public health systems for the timely detection and diagnosis of arboviruses and the lack of built-in national surveillance systems [24,25]. Overlapping and undifferentiated symptoms of arboviruses, coupled with a limited diagnostic capacity in Kenya, contribute to their underdiagnosis [4,26]. Moreoever, it has been shown that DENV disease can manifest differently in the African context compared to other parts of the world [27,28].

The main tools currently available to control DENV and CHIKV transmission and prevent outbreaks are surveillance and vector management [29]. Identifying the degree and areas of active transmission and risk factors that expose individuals to these viruses is vital for synthesizing interventions, educating communities, and informing public health policy and behavioral changes. The few studies that have investigated the seroprevalence and associated risk factors of arboviruses in Kenya have identified age, sex, occupation, mosquito preventive behavior, flooding, location, roof material, and presence of water collection around households to be associated with increased seroprevalence of DENV and CHIKV [13,30–33]. However, most of these studies are either non-prospective studies, only include participants from rural Kenya, or include only children if they are based in urban settings [6,13,15,30,32–34]. The aim of this study is to identify the risk factors associated with the seroprevalence and seroconversion of CHIKV and DENV infection in western and coastal Kenya among urban populations, including both adults and children. In addition, we describe the temporal patterns of seroconversion among cases. We hypothesized a significant prevalence and active circulation of DENV and CHIKV to be occurring in Kisumu and Ukunda that are not being reported with potentially more DENV prevalence on the coast and more CHIKV prevalence on the west.

## Methods

### Ethics statement

Approval for this study was obtained from the Institutional Review Boards of Stanford University (IRB #49683) and the Technical University of Mombasa Ethical Review Committee (TUM-ERC EXT/004/2019). It obtained a research license from the National Commission for Science, Technology & Innovation (NACOSTI/P/19/2574), Kenya. County administrators, both in Kisumu and Kwale counties, were officially informed about this research work before initiating field activities. Meetings (*barazas*) with the local residents and their administrators were also conducted to introduce and explain the objectives and planned activities. Written informed consent was obtained from all adults and parents or guardians of all participating children in our study. Verbal assent was also obtained from children aged 7 years and older**.**

### Study design and participants

This is a prospective longitudinal cohort study spanning over a period of slightly more than 2 years (December 2019-February 2022). To achieve 80% power in a 2-sided t-test with 0.05 significance level, using an estimate of 12% overall yearly CHIKV/DENV incidence and a conservative estimate of 4% incidence difference based on past studies, we planned to enroll at least 1,200 participants in each group. 4529 participants were recruited from 1,165 households at two urban sites in Kenya: Ukunda on the Coast (4°17′59.9994″S, 39°34′59.8794″E) and Kisumu in the West (0°5′15.22478″S, 34°46′22.3284″E). Each study site was stratified into 8 zones measuring approximately 200m x 200m, and consenting participants were enrolled from randomly selected households in each zone. Our study sites are considered urban based on their population density: Ukunda = 2000 people/km^2^; Kisumu = 15,000/km^2^) [35]. Participants were enrolled in the study between December 2019 and September 2020 and followed every 6 months until February 2022, when the study was completed. Both adults and children older than one year were recruited. Enrollment visits were recorded as baseline. Due to delays caused by the COVID-19 pandemic, the first follow-up was conducted 9 months following enrollment from October 2020 until March 2021. The second and third follow-ups were conducted from March 2021 until September 2021 and September 2021 until February 2022, respectively. The scheduled biannual follow-up visits were conducted at participants’ households by a study clinical officer. If any participants report a subjective fever within the last 7 days during the follow up interview, they were registered as a sick visit. If any participants fell ill in between the follow up time points, they were referred to a health center (Diani Health Centre in Ukunda and Migosi Sub-County Hospital in Kisumu) for treatment, if necessary, and registered as sick visits if they presented with a body temperature of ≥38°C. For those that could or did not report to the health care center, sick visits were conducted at their household by the study clinical officer. Blood samples collected at sick visits were all tested by RT-PCR to screen for acute DENV and CHIKV infections and the test results were notified to participants over a phone call. All sick visits were 0–7 days post-onset of fever. Due to limited resources, we only tested participants who reported fever by RT-PCR.

### Data collection

Questionnaires were administered to the consenting participants by trained study personnel during enrollment, scheduled follow-up visits, and sick visits to obtain demographic, household, and individual behavioral information. Physical and clinical examination was also collected during enrollment, scheduled follow-up visits, and sick visits. Demographic and behavioral questions were answered by adult participants themselves. Guardians answered on behalf of child participants. Questions assessing household characteristics were answered by the head of the household. Clinical history and physical examination information were also additionally collected at all timepoints. Venous blood samples were collected by a certified phlebotomist for serologic testing at enrollment, all three subsequent follow-ups, and all sick visits. Serum was separated from collected blood samples by centrifugation and stored at -70°C in the local laboratory at each study site. Each sample was labeled with a barcode and a unique identification number, maintaining participant anonymity. Separated serum, samples were then shipped to our laboratory at Stanford School of Medicine in the U.S., where they were stored at -70° C until tested. All serum samples were tested for anti-DENV and anti-CHIKV IgG using a previously described ELISA protocol [15,36]. To capture acute DENV and CHIKV infections among participants in real time, samples collected during sick visits were additionally tested by reverse transcriptase polymerase chain reaction (RT-PCR) in the local laboratories in Kenya [37,38]. All study data were uploaded to a secured database on REDCap hosted at the Stanford Center for Clinical Informatics.

### Statistical analysis

In this study, seropositivity was defined as testing positive on IgG ELISA assay at any time point, while a seroconversion was characterized as converting from seronegative (negative IgG ELISA result) to seropositive status (positive IgG ELISA result) during a subsequent follow-up time point within the study. Due to a notable study attrition rate during the COVID-19 pandemic and the presence of missing data in follow-up visits, this report relies on responses to risk factor questions obtained at enrollment for statistical analysis. Due to the restrictions on movement and social behavior during the COVID-19 pandemic, it is highly likely that risk factors remained relatively stable. Our analysis also excludes five individuals from the initial cohort who tested positive for DENV or CHIKV via PCR but did not demonstrate seroconversion in the subsequent follow-up ELISA results [39].

Categorical variable frequency variations were compared using chi-square analysis, while an independent t-test was employed to compare means for continuous variables. Univariate and multivariate logistic regression analyses were conducted to identify risk factors associated with DENV and CHIKV seropositivity and seroconversion, with detailed variables listed in Tables 3 and 4. Logistic regression played a pivotal role in predictive modeling, utilizing forward selection as a key technique for variable selection. This process involved iteratively adding variables to the model based on their individual contributions to improving model fit, as evaluated through statistical measures such as R-squared and AIC values. Additionally, a Kaplan-Meier survival analysis estimated the median survival time to seroconversion for both DENV and CHIKV. All data underwent analysis using SAS (r) Proprietary Software 9.4 (Copyright 2016 by SAS Institute Inc., Cary, NC, USA).

Various indices were also formulated to analyze key variables of interest. The original education categories encompassed a range from ‘no education’ to ‘college/university,’ which were subsequently consolidated into two broad classifications: ‘up to primary school’ (encompassing no education, some primary school, and completed primary school) and ‘secondary school and higher’ (encompassing some secondary school, completed secondary school, and college/university levels). Those who indicated having window screens ‘on some windows’ were collapsed with those that reported having no window screens to result in a two categories for window screens variable: ‘yes’ for households with window screens on all windows and ‘no’ for households with either some windows with screens or no windows with screens.

The Household Crowding Index (HCI) was derived by dividing the total number of residents per household by the total number of rooms (excluding the kitchen and bathrooms). The continuous HCI variable was further categorized into ‘not crowded’ (<3 residents per room) and ‘crowded’ (≥3 residents per room).For socioeconomic status (SES), a comprehensive index was established through Principal Component Analysis using demographic and household data, including marital status, household water source, sanitation, cooking fuel, asset ownership (e.g., bicycle, motor vehicle, electricity, microwave, mobile, motorcycle, radio, fridge, sofa, and TV), as well as floor and roofing material. SES ranks were determined using proc rank in SAS, classifying participants into low or high socioeconomic status. In addition, a Vector Control Behavior Index was created based on positive responses to various mosquito control methods assessed in the study, such as the use of insecticide-treated bed nets, mosquito coils, mosquito repellent, and house interior wall spraying. This index identified participants engaged in vector control behavior (S1 Table**)**.

## Results

Among the 4,529 participants, Ukunda (on the coast) comprised 44.4% (N = 2,008) of the study population, while Kisumu (on the west) accounted for 55.6% (N = 2,521). 1,555 households, 853 (54.9%) from Kisumu and 702 (45.1%) from Ukunda, were included in the study. Sex distribution showed 38.2% males and 61.8% females, with a median age of 21 years across both sites. Adults (58.6%) were slightly more represented in our study population than children (less than 16 years) (41.4%). There were no participants under the age of 1 year old per inclusion criteria. 869 cohort participants that presented for a sick visit during the study had PCR testing done. Our study population was well distributed across SES and education categories with a slightly higher representation of participants with higher SES (54.9%) and participants with education less than completed primary school (58.6%). Most of the study participants engaged in at least one mosquito control behavior (72.6%) and had less than three people per room (62.7%), no screens for their windows (59.6%), and no reported water collecting around the house (52.6%). After initial enrollment at baseline, we had a significant loss to follow up due to the COVID-19 pandemic and study shut down resulting in an overall 43.3% loss of participants by the end of 3^rd^ follow up. A comprehensive overview of the study population’s characteristics is detailed in **Table 1**.

**Table 1:** Descriptive statistics and demographics of study participants, Ukunda and Kisumu sites, Kenya, Dec 2019–Feb 2022.

Population Characteristics	Category	Overall (N = 4,529) N (%)	Ukunda (N = 2,008) N (%)	Kisumu (N = 2,521) N (%)
**Sex**	Male	1,728 (38.2)	751(37.4)	977 (38.8)
	Female	2,801 (61.8)	1,257(62.6)	1,544 (61.3)
**Age median in years (IQR)**		21 (9-33)	23 (10-36)	18 (8-31)
**Age group**	Child (>1, < 16 years)	1,877 (41.4)	1,286 (64.0)	1,155 (45.8)
	Adult (≥ 16)	2,652 (58.6)	722 (36.0)	1,366 (54.2)
**Study time points** ^a^	Baseline	4,534 (100)	2,013 (44.4)	2,521 (55.6)
	1st Follow-up	2,002 (100)	1,228 (61.3)	774 (38.7)
	2nd Follow-up	1,976 (100)	1,096 (55.5)	880 (44.5)
	3rd Follow-up	1,965 (100)	863 (43.9)	1,102 (56.1)
**SES** ^b^	Low	1,259 (45.1)	568 (54.7)	691 (39.4)
	High	1,534 (54.9)	471 (45.3)	1,063 (60.6)
**Level of education** ^b^	Primary school and below	2,502 (58.6)	1,159 (58.8)	1,343 (58.5)
	Secondary school and higher	1,765 (41.4)	813 (41.2)	952 (41.5)
**Household crowding** ^b^	Crowding	1,118 (37.3)	313 (28.1)	805 (42.7)
	No crowding	1,883 (62.7)	802 (71.9)	1,081 (57.3)
**Window screens** ^b^	Yes	1,420 (40.4)	856 (81.2)	564 (22.9)
	No	2,095 (59.6)	198 (18.8)	1,897 (77.1)
**Water collection** ^b^	Yes	1,615 (47.4)	484 (49.9)	1,131 (46.3)
	No	1,796 (52.6)	484 (50.1)	1,309 (53.7)
**Vector control behavior**	Yes	3,287 (72.6)	1,206 (60.1)	2,081 (82.6)
	No	1,242 (27.4)	802 (39.9)	440 (17.5)

^a^Number of participants at each time point, not additive as some participants were lost to follow up.

^b^Total N varies due to missing data.

**Fig 1:**
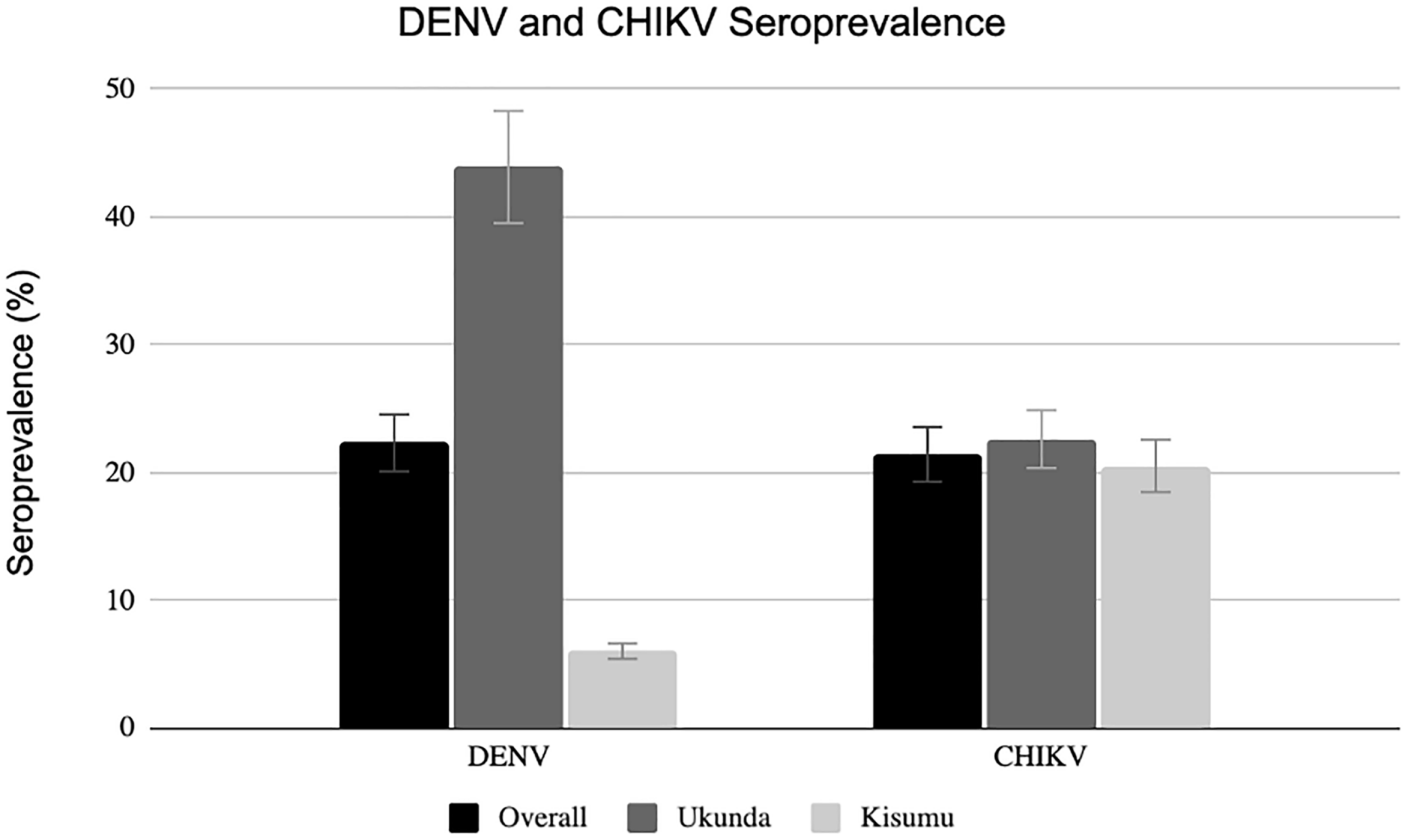
Seroprevalence of DENV and CHIKV among study participants in Kisumu and Ukunda, Kenya, Dec 2019 – Feb 2022.

The overall seropositivity for DENV and CHIKV at both study sites was 22.3% and 21.4%, respectively (**Fig 1**). Of the participants in Ukunda (coast), 882 (43.9%) were seropositive for DENV, which is markedly higher compared to Kisumu where 151 (6.0%) participants were DENV seropositve. In contrast, CHIKV seropositivity rates were relatively similar between the sites with 453 (22. 6%) seropositives in Ukunda compared to 516 (20.5%) seropositives in Kisumu. Of the total study population, 409 (9%) participants were seropositive for both viruses. Dual seropositivity was also notably higher in Ukunda compared to Kisumu with 333 (16.6%) and 76 (3.0%) participants seropositive for both DENV and CHIKV, respectively.

**Fig 2:**
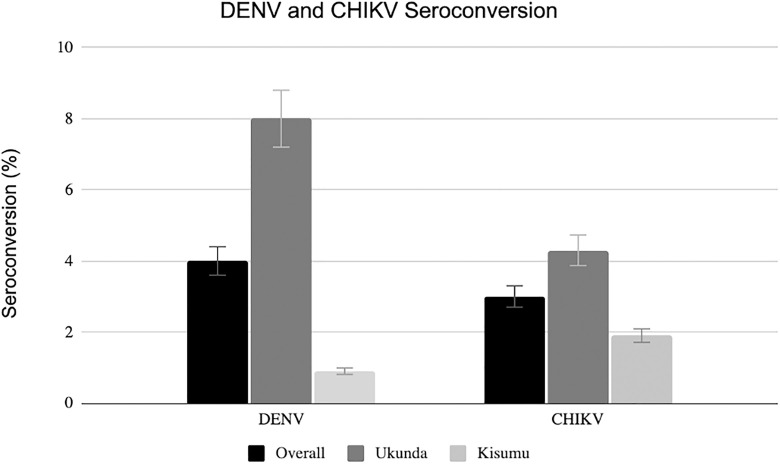
Seroconversion of DENV and CHIKV among study participants in Kisumu and Ukunda, Kenya, Dec 2019 – Feb 2022.

The overall seroconversion rate for DENV was 4.0% with 183 participants newly exposed to the virus and 3.0% for CHIKV seroconversion with 136 participants newly exposed during our 2-year study period (**Fig 2**). Of the 183 seroconverters for DENV, 161 (8%) were from Ukunda and 22 (0.9%) were from Kisumu (**Table 2**). Of the 136 seroconverters for CHIKV, 87 were from Ukunda and 49 were from Kisumu. The youngest participant to seroconvert was within the age range of 1–2 years. Of 869 samples tested by PCR from participants who presented with an acute febrile illness and had a sick visit, 31 (3.6%) tested positive for acute DENV infection, and 3 (0.4%) tested positive for acute CHIKV infection by RT-PCR. There is a slightly lower number of total samples tested for CHIKV than DENV by RT-PCR due to either insufficient sample or assay failure. The CT values for most DENV positive samples ranged from 27.11-33.23 with one weak positive at 40.39 while the CT values for CHIKV positive samples was 28.05-28.95. All of the sick visits with an acute infection were from Ukunda, majority female (65%) and 0–7 days post onset of fever.

**Table 2:** DENV and CHIKV IgG serology and RT-PCR test results among overall study population and individual study sites Ukunda and Kisumu, Kenya, Dec 2019–Feb 2022.

		Overall (N = 4,529) N (%)	Ukunda (N = 2,008) N (%)	Kisumu (N = 2,521) N (%)
**Anti-DENV IgG**	Positive	1,033 (22.8)	882 (43.9)	151 (6.0)
	Negative	3,496 (77.2)	1,126 (56.1)	2,370 (94.0)
**Anti-CHIKV IgG**	Positive	969 (21.4)	453 (22.6)	516 (20.5)
	Negative	3,560 (78.6)	1,555 (77.4)	2,005 (79.5)
**Dual seropositivity for both DENV and CHIKV**	Yes	409 (9.0)	333 (16.6)	76 (3.0)
	No	4,120 (91.0)	1,675 (83.4)	2,445 (97.0)
**Seroconversion** **Dengue**	Yes	183 (4.0)	161 (8.0)	22 (0.9)
	No	4,346 (96.0)	1,847 (92.0)	2,499 (99.1)
**Seroconversion Chikungunya**	Yes	136 (3.0)	87 (4.3)	49 (1.9)
	No	4,393 (97.0)	1,921 (95.7)	2,472 (98.1)
**Dengue PCR testing** ^a^	Positive	31 (3.6)	31 (10.8)	0 (0)
	Negative	838 (96.4)	257 (89.2)	580 (100)
**Chikungunya PCR testing** ^a^	Positive	3 (0.4)	3 (1.1)	0 (0)
	Negative	861 (99.7)	281 (98.9)	580 (100)

^a^PCR testing was limited to acutely febrile participants; thus, the total number is less than the full study population.

**Fig 3:**
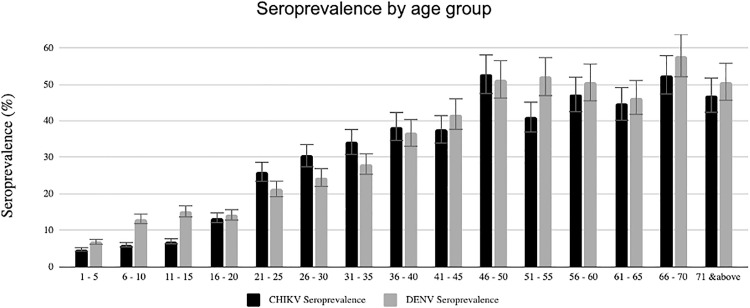
Distribution of DENV and CHIKV seroprevalence by age group of participants in the study.

The distribution of seropositivity across age groups, seen by 5-year increments, shows past exposure to DENV and CHIKV to be low (less than 20% seropositivity) among participants aged less than 20 years (**Fig 3**). Bimodal peaks occurred among individuals aged between 46 and 50 years and then again among individuals aged 70 and above.

### Risk factors associated with seroprevalence

Separate univariate and multivariate logistic regression analyses were performed with seropositivity for anti-DENV and anti-CHIKV IgG and seroconversion for anti-DENV and anti-CHIKV IgG positivity as primary outcomes. Our univariate logistic regression revealed a statistically significant association of residence in Ukunda on the coast, a secondary level or higher education, and older age with DENV seropositivity (p < 0.01). As expected, participants with no mosquito vector control behavior were also more likely to be seropositive for DENV (p < 0.01). Interestingly, households that had less crowding were also more likely to be seropositive for anti-DENV IgG (p < 0.01). Additionally, having window screens in the household was found to increase the odds of DENV seropositivity in the univariate analysis. However, after adjusting for other variables in our multivariate analysis, window screens were shown to have a protective effect against seropositivity though not statistically significant (OR= 0.86 [95% CI:0.61-1.21], p = 0.39). Female and older participants as well as participants with a secondary level or higher education were more likely to be seropositive for CHIKV (p < 0.01). Additionally, participants who had higher SES and water collecting around the house were more likely to be seropositive for CHIKV (p < 0.05).

To determine risk factors for seropositivity and seroconversion, a multivariate logistic regression model with stepwise selection was applied. After examining interactions between risk factors that may be related such as crowding and SES, a significant interaction between SES and level of education was found for the DENV seroconversion multivariate models. Consequently, the final model used to determine risk factors for DENV seroconversion included SES and not education. No other interactions were found in any of the other multivariate models. The multivariate logistic regression analysis found residing on the coast (OR=19.61 [95% CI:13.73-28.01], p=<0.01) and older age (OR=1.03 [95% CI:1.02-1.04], p=<0.01) to be associated with DENV seropositivity (Table 4a). Older age (OR= 1.05 [95% CI:1.04-1.06], p=<0.01), higher education (OR=1.37 [95% CI:1.1-1.69], p=<0.01), and crowding in the household (OR=1.3 [95% CI:1.04-1.62], p=<0.05) were associated with CHIKV seropositivity. The univariate and multivariate analyses for DENV and CHIKV seropositivity, including all variables, are presented in Tables 3 and 4, respectively.

**Table 3:** Univariate analysis between DENV and CHIKV seropositivity and selected risk factors.

		DENV seropositivity		CHIKV seropositivity	
		Unadjusted OR (95% CI)	p	Unadjusted OR (95% CI)	p
**Site**	Kisumu	Reference			
	Ukunda	12.29 (10.20-14.82)	<0.01*	1.13 (0.98-1.31)	0.09
**Sex**	Male	Reference			
	Female	1.11 (0.96-1.28)	0.16	1.21 (1.04-1.40)	<0.05*
**Age**		1.05 (1.04-1.05)	<0.01*	1.05 (1.05-1.06)	<0.01*
**Level of education**	Up to Primary School	Reference			
	Secondary and higher	1.32 (1.15-1.52)	<0.01*	1.78 (1.54-2.07)	<0.01*
**SES**	Low	Reference			
	High	0.83 (0.70-1.0)	0.05	0.82 (0.69-0.98)	<0.05*
**Household crowding** ^a^	No Crowding	Reference			
	Crowding	0.45 (0.37-0.56)	<0.01*	0.84 (0.737-1.06)	0.18
**Water collection**	Yes	Reference			
	No	0.89 (0.75-1.06)	0.19	0.84 (0.72-0.99)	<0.05*
**Window screens**	No	Reference			
	Yes	3.93 (3.3-4.69)	<0.01*	1.16 (0.99-1.36)	0.06
**Vector control behavior** ^b^	Yes	Reference			
	No	1.76 (1.52-2.04)	<0.01*	1.16 (1.0-1.36)	0.06

*Significant at p > 0.05 level

^a^Household crowding index was calculated as total number of residents per household divided by total number of rooms. Crowding was defined as having ≥3 persons/room

^b^Vector control behavior was defined as any use of insecticide-treated bed nets, mosquito coils, mosquito repellent, and interior house sprays

**Table 4:** Multivariate analysis between DENV and CHIKV seropositivity and selected risk factors.

		DENV seropositivity		CHIKV seropositivity	
		Adjusted OR (95% CI)	p	Adjusted OR (95% CI)	p
**Site**	Kisumu	Reference		Reference	
	Ukunda	19.61 (13.73-28.01)	<0.01*	0.93 (0.75-1.16)	0.53
**Age**		1.03 (1.03-1.04)	<0.01*	1.05 (1.04-1.06)	<0.01*
**Level of education** ^a^	Up to Primary School			Reference	
	Secondary and higher			1.37 (1.1-1.69)	<0.01*
**Water collection** ^a^	No	Reference			
	Yes	1.21 (0.91-1.61)	0.18		
**Window screens** ^a^	No	Reference			
	Yes	0.86 (0.61-1.21)	0.39		
**SES**	Low	Reference		Reference	
	High	1.35 (1.00-1.82)	0.05	0.82 (0.66-1.01)	0.06
**Household crowding** ^b^	No	Reference		Reference	
	Yes	0.74 (0.54-1.00)	0.05	1.3 (1.04-1.62)	<0.05*

*Significant at p > 0.05 level

^a^Greyed out cells indicate variables that were not included in the final analysis for the specific virus, either due to collinearity with another variable or lack of significance in univariate analysis.

^b^Household crowding index was calculated as total number of residents per household divided by total number of rooms. Crowding was defined as having ≥3 persons/room.

### Risk factors associated with seroconversion

Univariate analysis showed residing in Ukunda, older age, having window screens in the household, and no enagement in mosquito vector control behavior to be associated with DENV seroconversion. Consistent with DENV seropositivity, participants from Ukunda on the coast and of older age were more likely to seroconvert during the study period, becoming DENV seropositive. Participants who did not exhibit mosquito vector control behavior were also more likely to seroconvert for DENV. Similar to DENV seropositivity, having window screens was associated with DENV seroconversion in the univariate logistic regression analysis (p < 0.01) but when adjusting for other variables, became protective against seroconversion in the multivariate analysis (p < 0.01). Our univariate analysis also found residing in Ukunda, older age, and having window screens to be associated with CHIKV seroconversion. Similar to DENV seroconversion, participants from Ukunda and of older age were more likely to seroconvert for CHIKV (p < 0.01). The univariate analysis showed those who had window screens were more likely to seroconvert for CHIKV (p < 0.01), however, adjusting for other variables, having window screens showed a protective effect against seroconversion in the multivariate analysis (p < 0.01).

A multivariate logistic regression analysis with stepwise selection was also performed to determine risk factors for seroconversion. For DENV, the multivariate model revealed being in Ukunda on the coast (OR=25.28 [95% CI:12.38-51.63], p < 0.01) and high SES (OR=1.95 [95% CI:1.15-3.33], p < 0.05) increased the odds of seroconversion while having window screens (OR=0.42 [95% CI:0.23-0.75], p < 0.01) had a protective effect against seroconversion. The same risk factors were found associated with CHIKV seroconversion: being in Ukunda on the coast (OR= 31.93 [95% CI:14.92-68.36], p < 0.01) and high SES (OR=1.93 [95% CI:1.11-3.35], p < 0.05) increased odds of seroconversion as having window screens (OR=0.37 [95% CI:0.2-0.66], p < 0.01) showed a protective effect. The results of the univariate and multivariate analyses for DENV and CHIKV seroconversion, including all variables, are presented in Tables 5 and 6, respectively.

**Table 5:** Univariate analysis between DENV and CHIKV seroconversion and selected risk factors.

		DENV seroconversion		CHIKV seroconversion	
		Unadjusted OR (95% CI)	p	Unadjusted OR (95% CI)	p
Site*	Kisumu	Reference		Reference	
	Ukunda	9.87 (6.31-15.52)	<0.01*	2.28 (1.60-3.25)	<0.01*
**Sex**	Male	Reference		Reference	
	Female	1.07 (0.79-1.46)	0.66	1.35 (0.93-1.94)	0.1123
**Age**		1.02 (1.01-1.03)	<0.01*	1.03 (1.02-1.04)**	<0.01*
**Level of Education**	Up to Primary School	Reference		Reference	
	Secondary and higher	0.97 (0.71-1.32)	0.85	1.15 (0.81-1.63)	0.45
**SES**	Low	Reference		Reference	
	High	0.77 (0.53-1.12)	0.17	1.12 (0.71-1.77)	0.62
**Household crowding** ^a^	No Crowding	Reference		Reference	
	Crowding	0.77 (0.52-1.14)	0.2	0.79 (0.5-1.26)	0.32
**Water Collection**	Yes	Reference		Reference	
	No	1.22 (0.84-1.77)	0.3	0.82 (0.55-1.23)	0.34
**Window Screens**	No *	Reference		Reference	
	Yes	2.74 (1.88-3.99)	<0.01*	1.78 (1.21-2.65)	<0.01*
**Vector control behavior** ^b^	Yes	Reference		Reference	
	No	1.48 (1.09-2.02)	<0.05*	1.06 (0.73-1.55)	0.74

*Significant at p > 0.05 level

^a^Household crowding index was calculated as total number of residents per household divided by total number of rooms. Crowding was defined as having ≥3 persons/room

^b^Vector control behavior was defined as any use of insecticide-treated bed nets, mosquito coils, mosquito repellent, and interior house sprays.

**Table 6:** Multivariate analysis between DENV and CHIKV seroconversion and selected risk factors.

		DENV seroconversion		CHIKV seroconversion	
		Adjusted OR (95% CI)	p	Adjusted OR (95% CI)	p
**Site**	Kisumu	Reference	<0.01*	Reference	<0.01*
	Ukunda	25.28 (12.38-51.63)		31.93 (14.92-68.36)	
**Age**		1 (0.99 -1.02)	0.41	1.00 (0.99-1.02)	0.63
**Level of education**	Up to Primary			Reference	0.9
	Secondary and higher			0.96 (0.56-1.65)	
**SES**	Low	Reference	<0.05*	Reference	<0.05*
	High	1.95 (1.15-3.33)		1.93 (1.11-3.35)	
**Window screens**	No	Reference	<0.01*	Reference	<0.01*
	Yes	0.42 (0.23-0.75)		0.37 (0.20-0.66)	
**Household crowding** ^a^	No	Reference	0.56	Reference	0.61
	Yes	1.18 (1.18-0.68-2.04)		1.16 (0.66-2.04)	

*Significant at p > 0.05 level

^a^Crowding Index was calculated as total number of residents per household divided by total number of rooms. Crowding was defined as having ≥3 persons/room

### Survival analysis

The Kaplan-Meier curves depicting DENV seroconversions revealed that 2.5% (115 out of 4534) of study participants seroconverted against DENV within the first year. The overall median time to DENV seroconversion was 295 days (IQR = 0–515 days). Upon comparing sites, Ukunda exhibited a median time to DENV seroconversion of 406.5 days (IQR = 103–617), notably longer than Kisumu’s 169 days (IQR = 0–421).

In contrast, the Kaplan-Meier curves for CHIKV showed that 1.4% (62 out of 4534) of study participants seroconverted against CHIKV within the first year. The overall median time to CHIKV seroconversion was 290.5 days (IQR = 0–511 days). Site comparison indicated a median time to CHIKV seroconversion of 406.5 days (IQR = 103–617) in Ukunda and 169 days (IQR = 0–421) in Kisumu.

**Fig 4:**
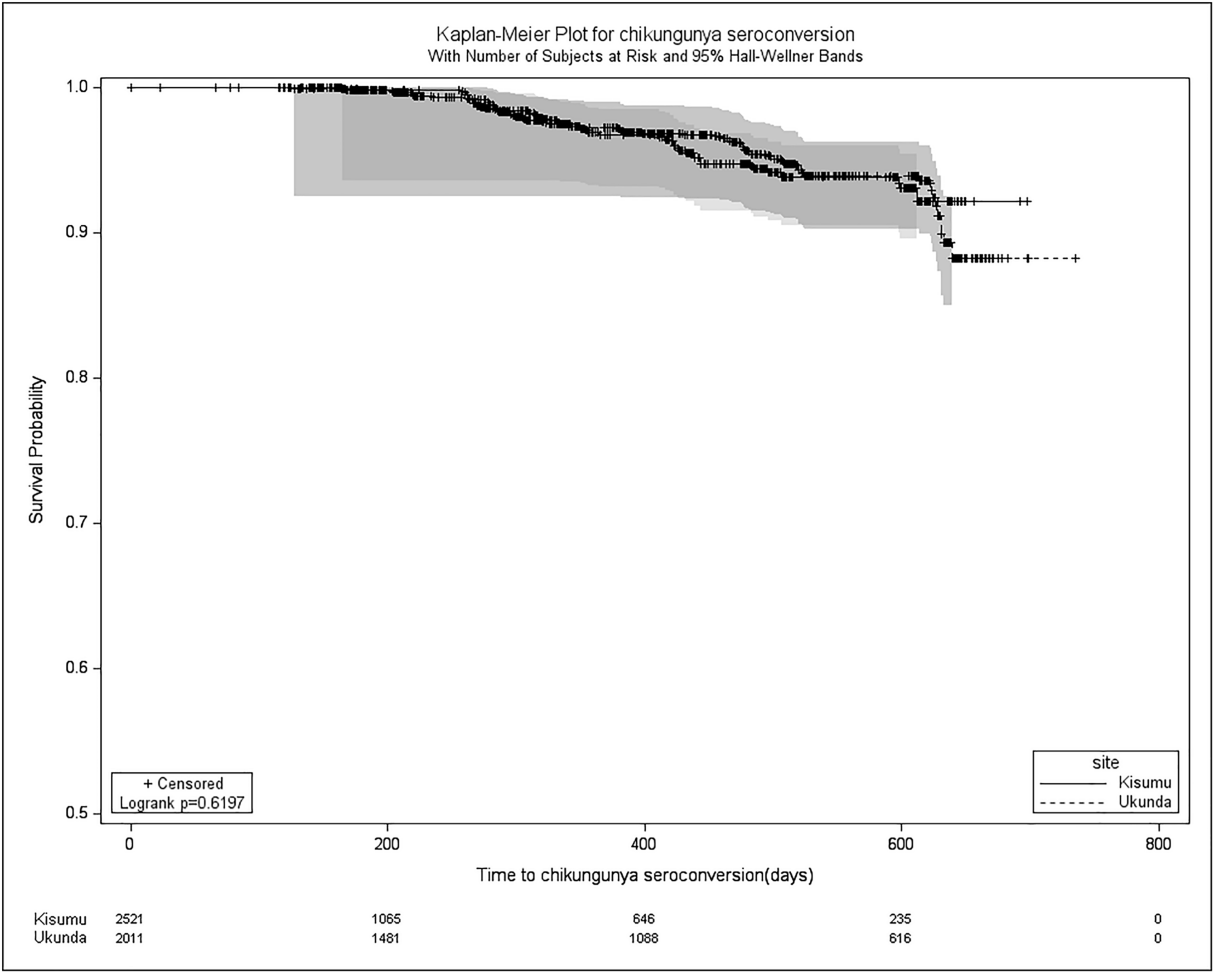
Kaplan Meier survival curves for new CHIKV seropositivity during the 2- year study period from Dec 2019 – Feb 2022.

**Fig 5:**
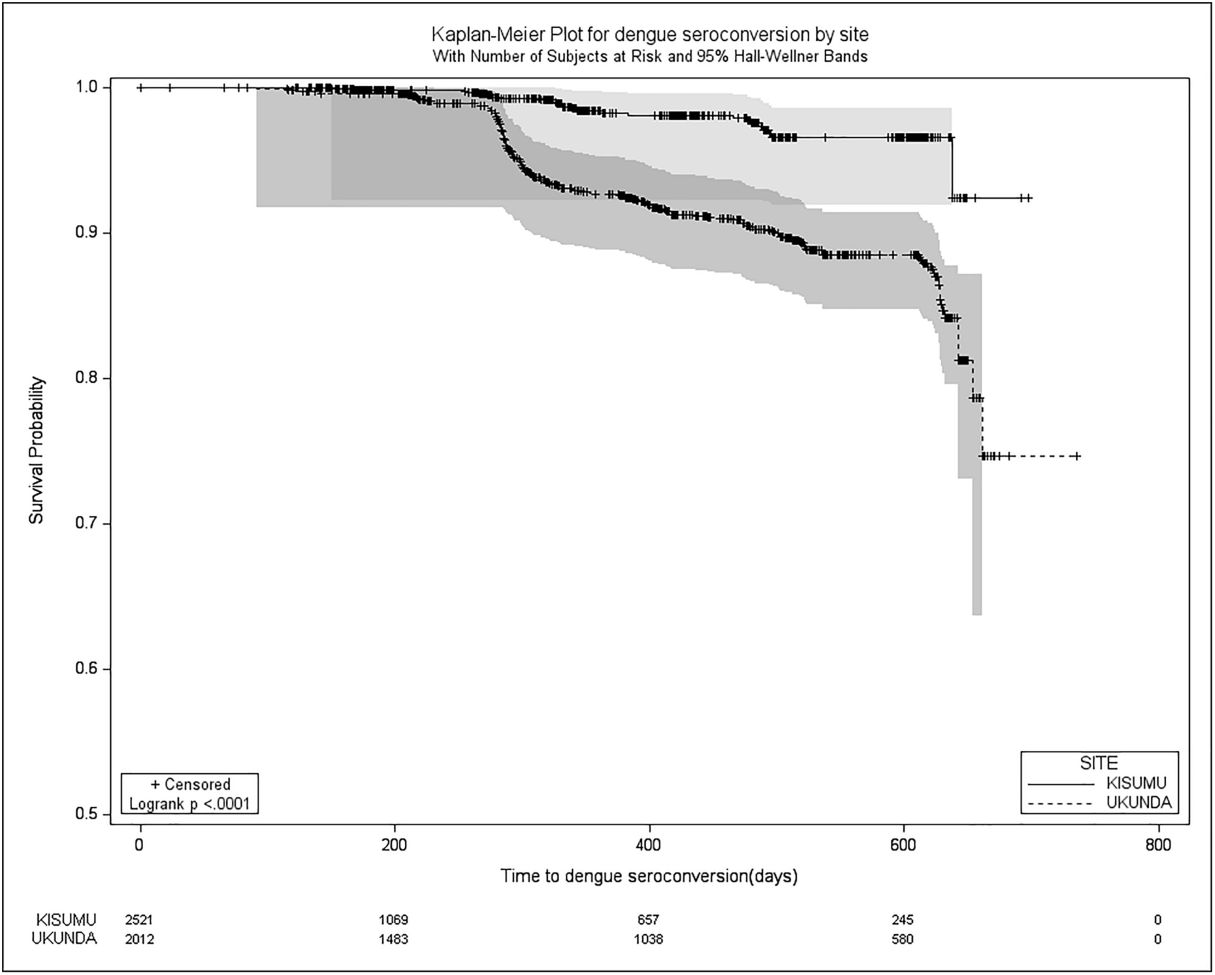
Kaplan Meier survival curves for new DENV seropositivity during the 2-year study period from Dec 2019 – Feb 2022..

It was unfeasible to establish the median survival time (time to seroconvert) for both DENV and CHIKV overall and by site, as less than 50% of the study population experienced seroconversion by the study’s conclusion. However, a disparity in survival was noted for DENV (log rank < 0.01) between sites, whereas no significant difference emerged for chikungunya (log rank = 0.93) in site-to-site comparison (**Figs 4** and **5**).

## Discussion

In this prospective longitudinal cohort study, we assessed the risk factors for and magnitude of DENV and CHIKV seroprevalence and seroconversion among urban populations in western and coastal Kenya. Our study confirms significant burden of DENV and CHIKV transmission in urban settings among both adults and children as well as active recent circulation of both viruses as seen by the newly exposed participants during the study. Adults were more likely to be exposed and seroconvert to either virus compared to children under the age of 16 years. The main risk factor for DENV seroprevalence was living on the coast and increasing age while increasing age, secondary level or higher education and crowding in the household were associated with CHIKV seroprevalence. The risk factors for seroconversion to both viruses were living on the coast, secondary level or higher education and not having window screens in the house.

In our study population, the overall seroprevalence for DENV and CHIKV were 22% and 21%, respectively, which is higher than previously reported seroprevalence for both viruses in Africa and Kenya. In 2021, a meta-analysis examined 43 studies from 2009-2020 and found the overall prevalence of DENV in Africa to be 14% and the prevalence during non-outbreak periods to be 3% [40]. Another meta-analysis study, including 76 studies, estimated the IgG seroprevalence of DENV to be 15.6% among apparently healthy individuals in Africa [41]. Specifically in Kenya, studies that included adult participants reported seroprevalence of DENV ranging from 1.1% to 12.5% [30,31,42–44]. However, one study that included children and adult participants in coastal Kenya found a higher DENV seroprevalence of 48%, although this study was restricted to two village clusters in Kwale County, our coastal study site [45]. Another meta-analysis examined 39 observational studies published between 2000 and 2017 and found the overall prevalence of CHIKV in Africa reported to be 16.4% during non-outbreak periods [46]. However, a meta-analysis of population-based studies reported an overall pooled seroprevalence of CHIKV to be 31% in Africa, but only included four studies from the region [47]. Specifically in Kenya, studies have reported the seroprevalence of CHIKV ranging from 2.1% to 22% [13,15,19,31,36,46,48,49]. The higher rate of seroprevalence we show in our study compared to prior studies makes it evident that urban populations in Kenya, including both adults and children, face significant exposures to both DENV and CHIKV, and may indicate growing transmission of these viruses in recent years.

In the past, arboviral outbreaks in Kenya have predominantly occurred on the coast including the 2004 CHIKV outbreak in Lamu Island, the 2013 and 2019 DENV outbreaks in Mombasa County, and the 2017 DENV outbreak in Malindi [50–52]. Our study shows there is still a heightened risk of seropositivity for DENV and CHIKV in this region of Kenya. In Ukunda on the coast, located just below Mombasa, nearly half of our study participants, including children, tested positive for anti-DENV IgG (44%). We also saw significant dual seropositivities to and active circulation of both DENV and CHIKV on the coast at higher rates than in the west. Additionally, even though past studies show significantly more seropositivity for CHIKV in the west than on the coast, we saw a slightly higher prevalence of CHIKV in Ukunda (23%) than in Kisumu on the west (21%) [15]. This could indicate a rise in CHIKV circulation on the coast following the 2017 and 2018 CHIKV outbreak in Mombasa county [53]. The higher seroprevalence seen in the Ukunda could be due to Ukunda being a major commercial port leading to introductions of pathogens from other parts of the world. Moreover, the proximity to natural water body, warmer tempreatures and high humidity of the coast contributes to the abundance vectors and transmissions of arboviruses. Within western Kenya, consistent with prior findings, our study results also show more CHIKV prevalence than DENV [15,30]. This was also reflected in our seroconversion results that show more active circulation of CHIKV in the west during the time of our study with more individuals newly exposed to CHIKV than DENV. It is known that DENV outbreaks on the coast of Kenya have arisen from strains of the virus that had already been circulating for years [54,55]. Without proper and much-needed active surveillance, vector control interventions, and awareness, the arboviruses that are currently circulating pose a threat to future outbreaks that should not be ignored.

Univariate and multivariate analyses were conducted to identify risk factors for both seropositivity and seroconversion to both DENV and CHIKV. Residence on the coast increased the odds of both seropositivity and seroconversion, although it was not statistically significant for CHIKV seropositivity, which is logical given the comparable CHIKV seroprevalence in the west and the coast. This result was also reflected in the multivariate analysis where residing on the coast was a risk factor for DENV seropositivity and seroconversions to DENV and CHIKV, highlighting the need for vector control and other interventions on the coast. Increasing age was identified as a risk factor for both seropositivity and seroconversion to DENV and CHIKV. As reported in other studies, adults have lived longer and tend to move around more than children, increasing their probability exposure to arboviruses, and consequently, their risk of seropositivity [40,56–61]. The univariate analysis showed that women were 21% more likely to be seropositive for CHIKV. A previous study had a similar observation where women were more likely to be exposed to alphaviruses on the coast of Kenya [36]. This observation was attributed to the cultural roles women occupy where they spend considerable time around homesteads where anthropophilic *Aedes* mosquitoes are prevalent [36]. However, controlling for other variables, sex did not persist as a significant risk factor in our multivariate analyses.

Education level was associated with seropositivity for DENV and CHIKV in our univariate analyses. While lower education has historically been associated with increased seropositivity for arboviruses, we found those with higher education are also at risk of seropositivity for DENV and CHIKV [62]. Participants with secondary level or higher education were more likely to be seropositive to CHIKV even after controlling for other variables in our multivariate analysis. While this is surprising, it is not without precedent [6]. A study looking at risk factors associated with arboviral seropositivity in rural Kenyan adults found literacy significantly associated with both DENV and CHIKV seropositivity [33]. Another study from Tanzania also looked at risk factors for arboviral seropositivity and found secondary education to be associated with CHIKV seropositivity compared to lower education although it was not statistically significant [63]. Similar to education, we also found a paradoxical association between SES and seroconversion. Previously, arboviral infections have been associated with low socioeconomic status in Africa and other parts of the world [59,64,65]. In our study, after controlling for other variables, higher SES was significantly associated with DENV and CHIKV seroconversion, which was also seen in a similar study conducted from 2014-2018 at our study sites in Kisumu and Ukunda [6]. There could be several explanation for this phenomena that put participants with higher SES and education at higher risk of seropositivity for DENV and CHIKV. Individuals with higher SES and education are more likely to travel and engage in outdoor activities, such recreation near standing water bodies, which can increase their exposure to *Aedes* mosquitos. Moreover, these individuals may work in areas with higher mosquito density such as outdoors or live in residences that are allow for mosquito breeding. These same behaviors may have also exposed participants who were seronegative at enrollment to new infections causing themt o seroconvert during the study. It is important to note, however, that within the context of our study, high SES represents relative wealth within our study sites that still are low-resource settings and does not refer to absolute wealth. Crowding in the household was included in our analyses as a surrogate marker for poverty and since *Aedes* mosquitoes can feed on multiple people within one gonotrophic cycle, the risk of infection can rise with overcrowding [66]. Participants with crowding in the household were more likely to be CHIKV seropositive, although crowding in the household showed a non-significant protective effect against DENV seropositivity. We also found crowding as a risk factor for DENV and CHIKV seroconversion, though not statistically significant. One of the prominent vector control practices involves installing window screens in households to prevent the entry of vector mosquitoes. As expected, participants who had window screens in their households were at a lower risk of seroconversion to both DENV and CHIKV.

Our study had several limitations. Due to the COVID-19 pandemic, a significant number of our participants were lost to follow-up and we chose to enroll new participants at our first follow-up time point to increase study numbers, 9 months after the original enrollment. For the new enrollments, the same procedures as the initial enrollment were followed. These individuals had a shorter time in our study and may have biased our results to lower seroconversion rates. Additionally, due to incomplete responses from participants, our follow-up dataset had some missing data resulting in the use of responses at enrollment. Cross-reactivity among flaviviruses and among alphaviruses has been shown to occur in serology assays. The detection of anti-DENV and anti-CHIKV IgG antibodies in our study by our in house ELISAs might be affected by such cross-reactivity from other circulating alphaviruses and flaviviruses. Moreover, due to our study size and limited resources, we could not perform PRNT to confirm specific virus; though our prior studies have demonstrated repeatedly that CHIKV and DENV are the most prominent alpha- and flaviviruses at our sties. Lastly, our testing protocol was constrained by capacity limitations, necessitating the focus for acute infection testing by RT-PCR solely on febrile participants.

In conclusion, this study presents evidence of significant seropositivity for DENV and CHIKV among urban populations in western (Kisumu) and coastal (Ukunda) Kenya, as well as the ongoing active circulation of these viruses. We also show that even individuals with secondary level or higher education and higher socioeconomic status are at risk of seropositivity for DENV and CHIKV. In coastal Kenya, we document higher rates of CHIKV prevalence than previously reported as well as continued active transmission of DENV, highlighting the need to raise awareness and enact interventions on the coast. Arboviruses continue to pose a significant threat to human health in Kenya, calling for nationwide active surveillance, enhanced vector control efforts, and community-engaged initiatives to increase awareness and agency among households to prevent exposure. The varying levels of seropositivity for DENV and CHIKV seen between western and coastal Kenya also highlight the importance of having interventions that are site-specific and cater to the specific needs of different communities. Lastly, our results emphasize the need to study, surveil, and control arboviral transmissions in African populations even during non-outbreak periods.

## Supporting information

S1 Table Indices, categories created, and the predictors included. (DOCX)

S2 Table DENV and CHIKV seroprevalence distribution. (DOCX)

S3 Table DENV and CHIKV seroconversion distribution. (DOCX)

S4 Table Distribution of DENV and CHIKV seropositivity and across age groups among study population. (DOCX)
