# Supplementary material for: Risk factors associated with dengue and chikungunya seroprevalence and seroconversion among urban populations in western and coastal Kenya
Source: PLoS Negl Trop Dis. 2025 Nov 24;19(11):e0013740. doi: 10.1371/journal.pntd.0013740 (PMC12747438; doi:10.1371/journal.pntd.0013740)
Supplement: S1 Table — (DOCX) [file pntd.0013740.s001.docx]

**S1 Table: Indices, categories created, and the predictors included**

| Indices created | Predictors included | Categories created |
| --- | --- | --- |
| Education | No education  Some primary school  Completed primary school  Some secondary school  Completed secondary school  College/university  Technical college | - Up to primary school - Secondary school and higher |
| Household crowding index | Total number of residents per household divided by total number of rooms (excluding the kitchen and bathrooms) | - <3 persons/room=not crowded - ≥3 persons/room=crowded |
| Socioeconomic status index | Marital status, household water source, sanitation, cooking fuel, asset ownership (bicycle, motor vehicle, electricity, microwave, mobile phone, motorcycle, radio, fridge, sofa, and TV), floor and roofing material | - Low SES - High SES |
| Vector control behavior | Use of any of these methods: insecticide-treated bed nets, mosquito coils, mosquito repellent, and spraying of the house interior walls | - Yes - No |
| Window screens | No window screens= No  Yes, but some windows= No  Yes, all windows= Yes | - Yes - No |
