# Supplementary material for: Risk factors associated with dengue and chikungunya seroprevalence and seroconversion among urban populations in western and coastal Kenya
Source: PLoS Negl Trop Dis. 2025 Nov 24;19(11):e0013740. doi: 10.1371/journal.pntd.0013740 (PMC12747438; doi:10.1371/journal.pntd.0013740)
Supplement: S2 Table — (DOCX) [file pntd.0013740.s002.docx]

**S2 Table: Descriptive statistics of DENV and CHIKV seroprevalence distribution across risk factors**

| **Variable** | **Dengue seropositivity** | | | | **p** | **Chikungunya seropositivity** | | | **p** |
| --- | --- | --- | --- | --- | --- | --- | --- | --- | --- |
|  |  | **Positive**  **N (%)** | **Negative**  **N (%)** | **Total N** |  | **Positive**  **N (%)** | **Negative**  **N (%)** | **Total N** |  |
| **Site** | Kisumu | 151 (6.0) | 2,370 (94.0) | 2,521 | <0.01 | 516 (20.5) | 2,005 (79.5) | 2,522 | 0.09 |
|  | Ukunda | 882 (43.9) | 1,126 (56.1) | 2,008 |  | 453 (22.6) | 1,555 (77.4) | 2,008 |  |
| **Sex** | Female | 658 (23.5) | 2,143 (76.5) | 2,801 | 0.11 | 633 (22.6) | 2,168 (77.4) | 2,802 | <0.05 |
|  | Male | 375 (21.7) | 1,353 (78.3) | 1,728 |  | 336 (19.4) | 1,392 (80.6) | 1,728 |  |
| **Age median in years (IQR )** |  | 32 (20-45) | 17 (8-30) |  |  | 33 (24-45) | 16.0 (7.0-30.0) |  |  |
| **Age Group** | Adult (≥16) | 818 (30.8) | 1,834 (69.2) | 2,652 | <0.01 | 859 (32.4) | 1,793 (67.6) | 2,652 | <0.01 |
|  | Child (<16) | 215 (11.5) | 1,662 (88.5) | 1,877 |  | 110 (5.9) | 1,767 (94.1) | 1,877 |  |
| **Level of education** | Primary and below | 540 (21.6) | 1,962 (78.4) | 2,502 | <0.01 | 435 (17.4) | 2,067 (82.6) | 2,502 | <0.01 |
|  | Secondary school and higher | 471 (26.7) | 1,294 (73.3) | 1,765 |  | 482 (27.3) | 1,283 (72.7) | 1,765 |  |
| **SES** | High | 305 (19.9) | 1,229 (80.1) | 1,534 | 0.05 | 318 (20.7) | 1,216 (79.3) | 1,534 | <0.05 |
|  | Low | 289 (23.0) | 970 (77.0) | 1,259 |  | 304 (24.2) | 955 (75.8) | 1,259 |  |
| **Household crowding** | Crowded | 151 (13.5) | 967 (86.5) | 1,118 | 0.33 | 229 (20.5) | 889 (79.5) | 1,118 | <0.01 |
|  | Not crowded | 481 (25.5) | 1,402 (74.4) | 1,883 |  | 425 (22.6) | 1,458 (77.4) | 1,883 |  |
| **Water collection** | No | 330 (18.4) | 1,466 (81.6) | 1,796 | 0.3449 | 390 (21.70 | 1,406 (78.3) | 1,796 | 0.20 |
|  | Yes | 325 (20.1) | 1,290 (79.9) | 1,615 |  | 400 (24.8) | 1,215 (75.2) | 1,615 |  |
| **Window screens** | No | 232 (11.1) | 1,863 (88.9) | 2,095 | <0.01 | 463 (22.1) | 1,632 (77.9) | 2,095 | <0.01 |
|  | Yes | 467 (32.9) | 953 (67.1) | 1,420 |  | 346 (24.4) | 1,074 (75.6) | 1,420 |  |
| **Vector control behavior** | No | 378 (30.4) | 864 (69.6) | 1,242 | 0.74 | 289 (23.3) | 953 (76.7) | 1,242 | <0.01 |
|  | Yes | 655 (19.9) | 2,632 (80.1) | 3,287 |  | 680 (20.7) | 2,607 (79.3) | 3,287 |  |
