# Supplementary material for: Risk factors associated with dengue and chikungunya seroprevalence and seroconversion among urban populations in western and coastal Kenya
Source: PLoS Negl Trop Dis. 2025 Nov 24;19(11):e0013740. doi: 10.1371/journal.pntd.0013740 (PMC12747438; doi:10.1371/journal.pntd.0013740)
Supplement: S3 Table — (DOCX) [file pntd.0013740.s003.docx]

**S3 Table: Descriptive statistics of DENV and CHIKV seroconversion distribution across risk factors**

| **Variables** | **Dengue seroconversion** | | | | **p** | **Chikungunya seroconversion** | | | **p** |
| --- | --- | --- | --- | --- | --- | --- | --- | --- | --- |
|  |  | **Yes**  **N (%)** | **No**  **N(%)** | **Total N** |  | **Yes**  **N(%)** | **No**  **N(%)** | **Total N** |  |
| **Site** | Kisumu | 22 (0.9) | 2,499 (99.1) | 2,521 | <0.01 | 49 (1.9) | 2,472 (98.1) | 2,521 | <0.01 |
|  | Ukunda | 161 (8.0) | 1,847 (92.0) | 2,008 |  | 87 (4.3) | 1,921 (95.7) | 2,008 |  |
| **Sex** | Female | 116 (4.1) | 2,685 (95.9) | 2,801 | 0.66 | 93 (3.3) | 2,708 (96.7) | 2,801 | 0.11 |
|  | Male | 67 (3.9) | 1,661 (96.1) | 1,728 |  | 43 (2.5) | 1,685 (97.5) | 1,728 |  |
| **Age median in years (IQR)** |  | 26 (12-41) | 20.0 (9-33) |  |  | 30 (16.0-44.5) | 20 (9-33) |  |  |
| **Age group** | Child | 59 (3.1) | 1,818 (96.9) | 1,877 | <0.01 | 33 (1.8) | 1,844 (98.2) | 1,877 | <0.05 |
|  | Adult | 124 (4.7) | 2,528 (95.3) | 2,652 |  | 103 (3.9) | 2,549 (96.1) | 2,652 |  |
| **Level of education** | Primary and below | 105 (4.20 | 2,397 (95.8) | 2,502 | 0.85 | 72 (2.9) | 2,430 (97.1) | 2,502 | 0.44 |
|  | Secondary school and higher | 72 (4.1) | 1,693 (95.9) | 1,765 |  | 58 (3.3) | 1,707 (96.7) | 1,765 |  |
| **SES** | High | 57 (3.7) | 1,477 (96.3) | 1,534 | 0.17 | 45 (2.9) | 1,489 (97.1) | 1,534 | 0.62 |
|  | Low | 60 (4.8) | 1,199 (95.2) | 1,259 |  | 33 (2.6) | 1,226 (97.4) | 1,259 |  |
| **Household crowding** | Crowded | 38 (3.4) | 1,080 (96.6) | 1,118 | 0.18 | 27 (2.4) | 1,091 (97.6) | 1,118 | 0.20 |
|  | Not crowded | 82 (4.4) | 1,801 (95.6) | 1,883 |  | 57 (3.0) | 1,826 (97.0) | 1,883 |  |
| **Water collection** | no | 66 (3.7) | 1,730 (96.3) | 1,796 | <0.05 | 48 (2.7) | 1,748 (97.3) | 1,796 | 0.30 |
|  | yes | 49 (3.0) | 1,566 (97.0) | 1,615 |  | 52 (3.2) | 1,563 (96.8) | 1,615 |  |
| **Window screens** | No | 44 (2.1) | 2,051 (97.9) | 2,095 | 0.11 | 47 (2.2) | 2,048 (97.8) | 2,095 | <0.01 |
|  | Yes | 79 (5.6) | 1,341 (94.4) | 1,420 |  | 56 (3.9) | 1,364 (96.1) | 1,420 |  |
| **Vector control behavior** | No | 65 (5.2) | 1,177 (94.8) | 1,242 | <0.05 | 39 (3.1) | 1,203 (96.9) | 1,242 | 0.06 |
|  | Yes | 118 (3.6) | 3,169 (96.4) | 3,287 |  | 97 (3.0) | 3,190 (97.0) | 3,287 |  |
