# Supplementary material for: Risk factors associated with dengue and chikungunya seroprevalence and seroconversion among urban populations in western and coastal Kenya
Source: PLoS Negl Trop Dis. 2025 Nov 24;19(11):e0013740. doi: 10.1371/journal.pntd.0013740 (PMC12747438; doi:10.1371/journal.pntd.0013740)
Supplement: S4 Table — (DOCX) [file pntd.0013740.s004.docx]

**S4 Table: Distribution of DENV and CHIKV seropositivity and across age groups among study population**

|  | **DENV seropositivity** | |  | **CHIKV seropositivity** | |  |
| --- | --- | --- | --- | --- | --- | --- |
| **Age group** | **Yes** | **No** | **Total N** | **Yes** | **No** | **Total N** |
| **1 - 5** | 46 (6.8%) | 628 (93.2%) | 674 | 32 (4.7%) | 642 (95.3%) | 674 |
| **6 - 10** | 85 (13.1%) | 562 (86.9%) | 647 | 39 (6.0%) | 608 (94.0%) | 647 |
| **11 - 15** | 85 (15.2%) | 475 (84.8%) | 560 | 39 (7.0%) | 521 (93.0%) | 560 |
| **16 - 20** | 54 (14.2%) | 326 (85.8%) | 380 | 51 (13.4%) | 329 (86.6%) | 380 |
| **21 - 25** | 100 (21.3%) | 369 (78.7%) | 469 | 122 (26.0%) | 347 (74.0%) | 469 |
| **26 - 30** | 102 (24.5%) | 315 (75.5%) | 417 | 127 (30.5%) | 290 (69.5%) | 417 |
| **31 - 35** | 116 (28.2%) | 296 (71.8%) | 412 | 141 (34.2%) | 271 (65.8%) | 412 |
| **36 - 40** | 105 (36.7%) | 181 (63.3%) | 286 | 110 (38.5%) | 176 (61.5%) | 286 |
| **41 - 45** | 90 (41.9%) | 125 (58.1%) | 215 | 81 (37.7%) | 134 (62.3%) | 215 |
| **46 - 50** | 74 (51.4%) | 70 (48.6%) | 144 | 76 (52.8%) | 68 (47.2%) | 144 |
| **51 - 55** | 61 (52.1%) | 56 (47.9%) | 117 | 48 (41.0%) | 69 (59.0%) | 117 |
| **56 - 60** | 46 (50.5%) | 45 (49.5%) | 91 | 43 (47.3%) | 48 (52.7%) | 91 |
| **61 - 65** | 26 (46.4%) | 30 (53.6%) | 56 | 25 (44.6%) | 31 (55.4%) | 56 |
| **66 - 70** | 22 (57.9%) | 16 (42.1%) | 38 | 20 (52.6%) | 18 (47.4%) | 38 |
| **71 & above** | 207 (50.7%) | 201 (49.3%) | 408 | 192 (47.1%) | 216 (52.9%) | 408 |
